# Supplementary material for: Regulatory T Cell Apoptosis during Preeclampsia May Be Prevented by Gal-2
Source: Int J Mol Sci. 2022 Feb 7;23(3):1880. doi: 10.3390/ijms23031880 (PMC8836599; doi:10.3390/ijms23031880)
Supplement: Supplementary file 1 [file ijms-23-01880-s001.zip › ijms-1575157 - supplementary revised.pdf]

Supplementary Material

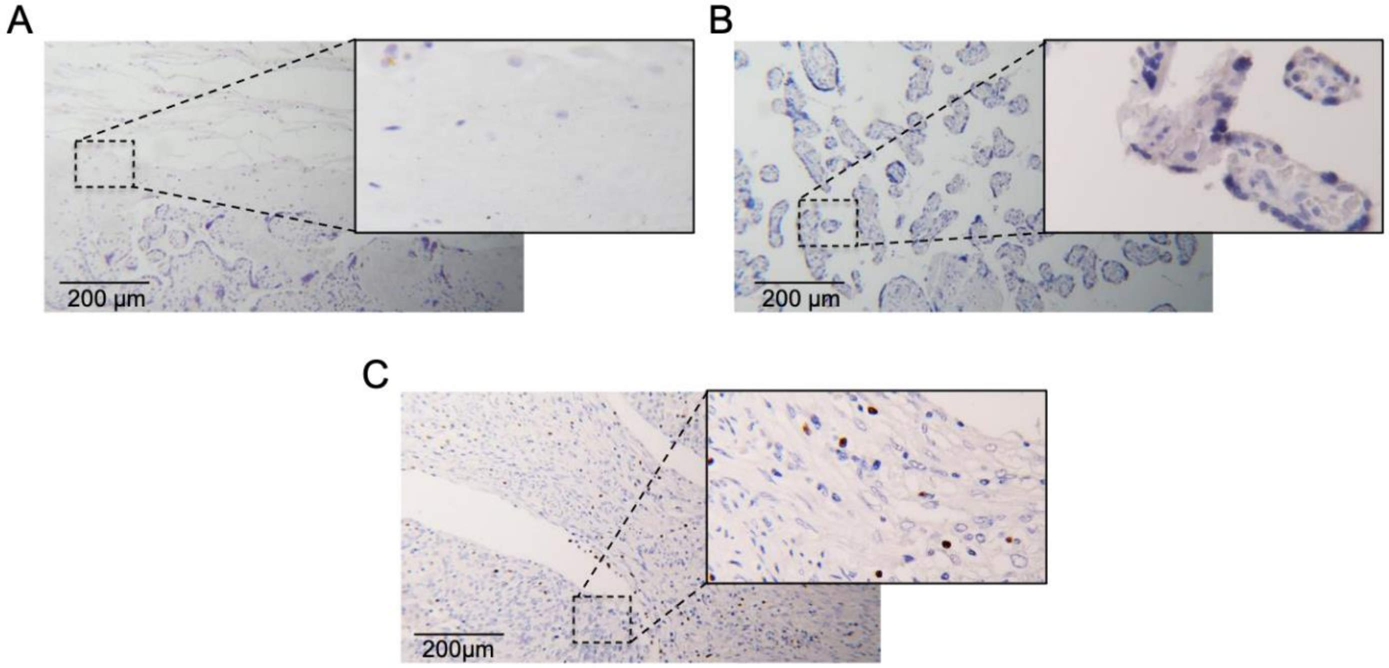

Figure S1. Corresponding negative controls of the decidua (**A**) and the syncytium (**B**) of placental tissue, as well as a positive control of FoxP3 conducted in cervical cancer tissue (**C**).

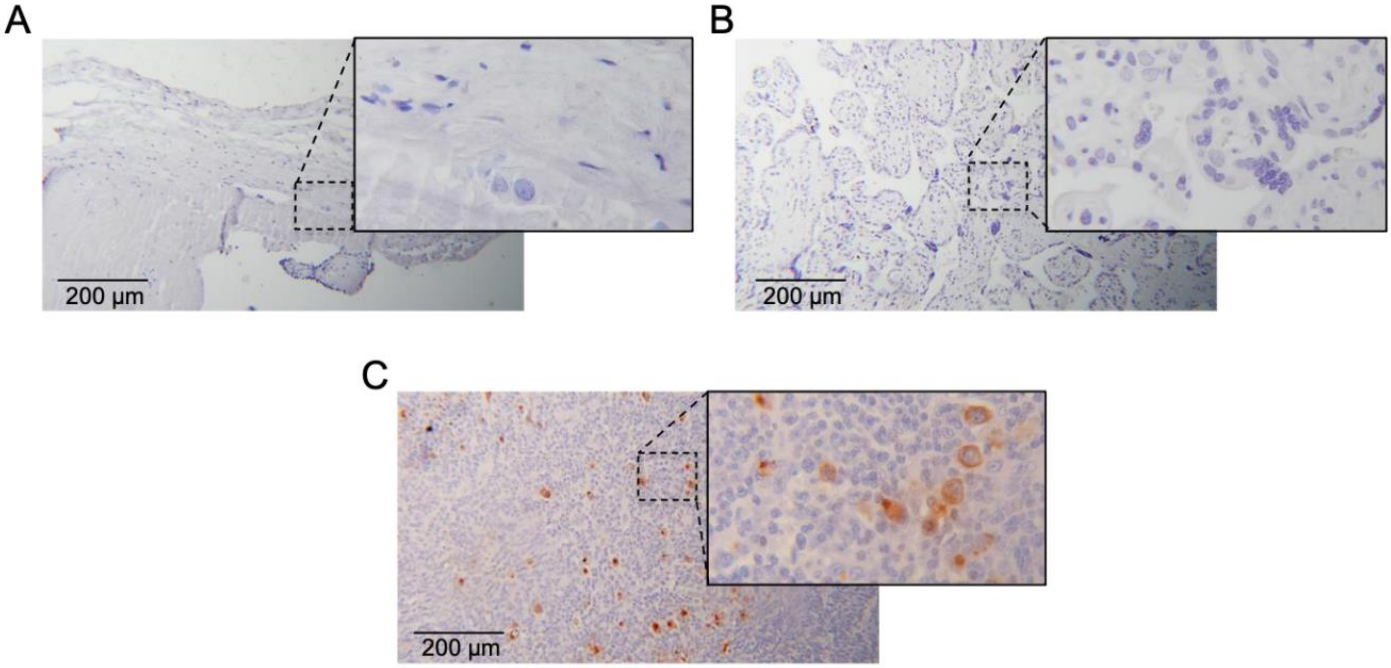

Figure S2. Corresponding negative controls of the decidua (**A**) and the syncytium (**B**) of placental tissue, as well as a positive control of CCL22 conducted in thymus tissue (**C**).

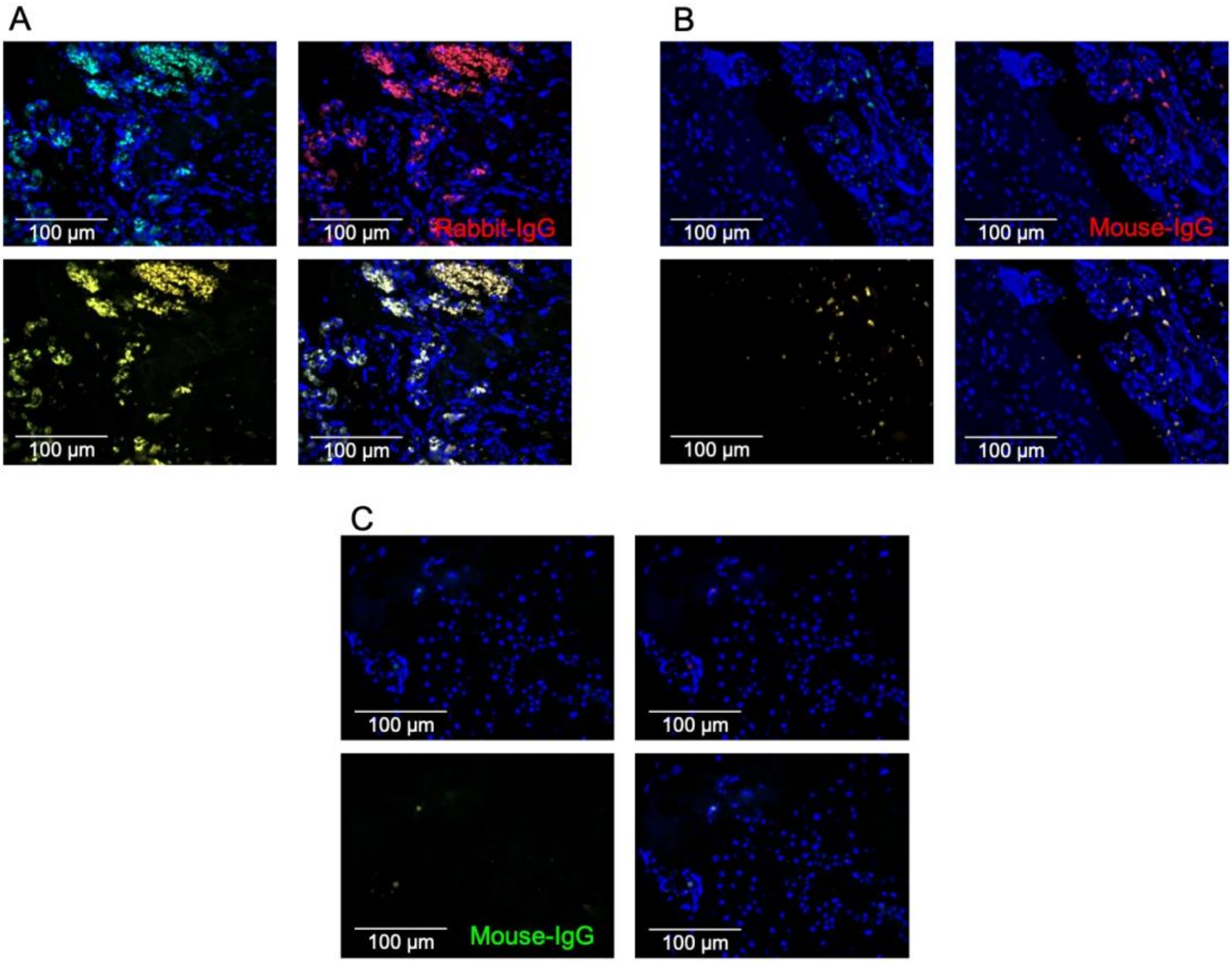

Figure S3. Corresponding immunofluorescencial negative controls. Since TUNEL was already pre-labeled, negative controls were only conducted for the Cy3-labeled-goat-anti-rabbit-IgG (red) used for the staining of CCL22 (**A**), the Cy3-labeld-goat-anti-mouse-IgG (red) used for the staining of FoxP3 (**B**) and the AlexaFluor-488-labeled-goat-anti-mouse-IgG (green) used for the staining of CK7 (**C**). All pictures are presented as DAPI/Cy2 in the upper left, DAPI/Cy3 in the upper right, Cy2/Cy3 in the lower left and merge of all channels in the lower right.

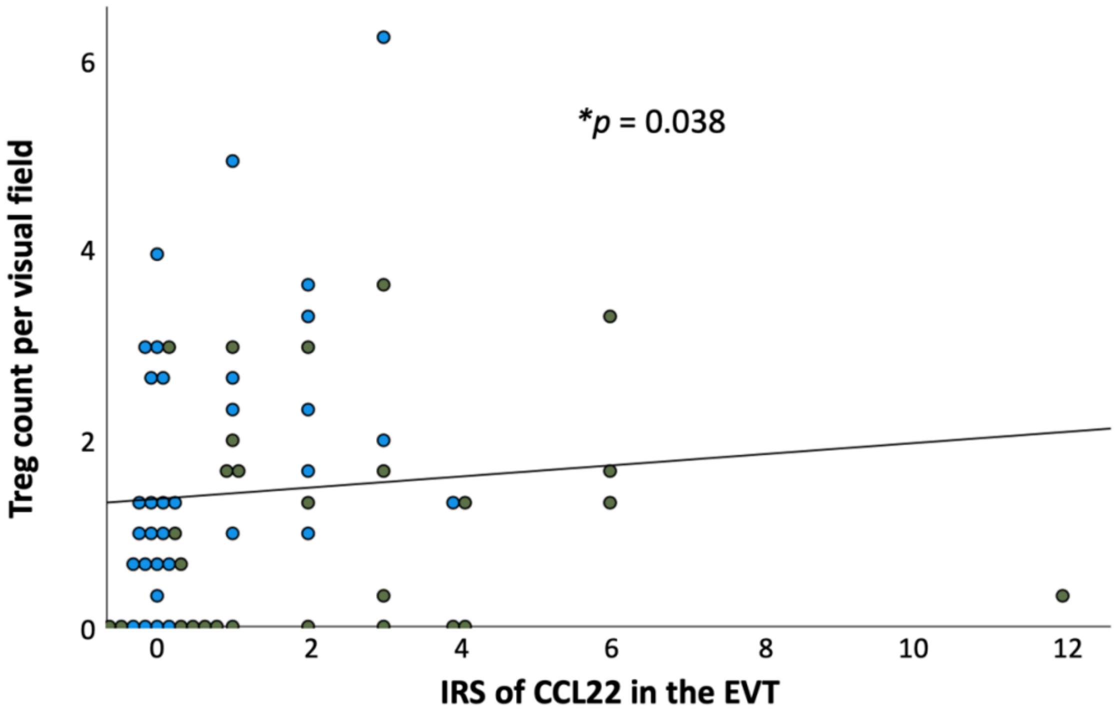

Figure S4. Visualization of the correlation of the expression of CCL22 in the EVT and the number of Tregs per visual field. Correlation was calculated using the Spearman-Rho-Correlation-Test (r = 0.256, p = 0.042). The regression line refers to the total collective. The 34 control placentas are shown in blue, the 32 PE placentas in green.

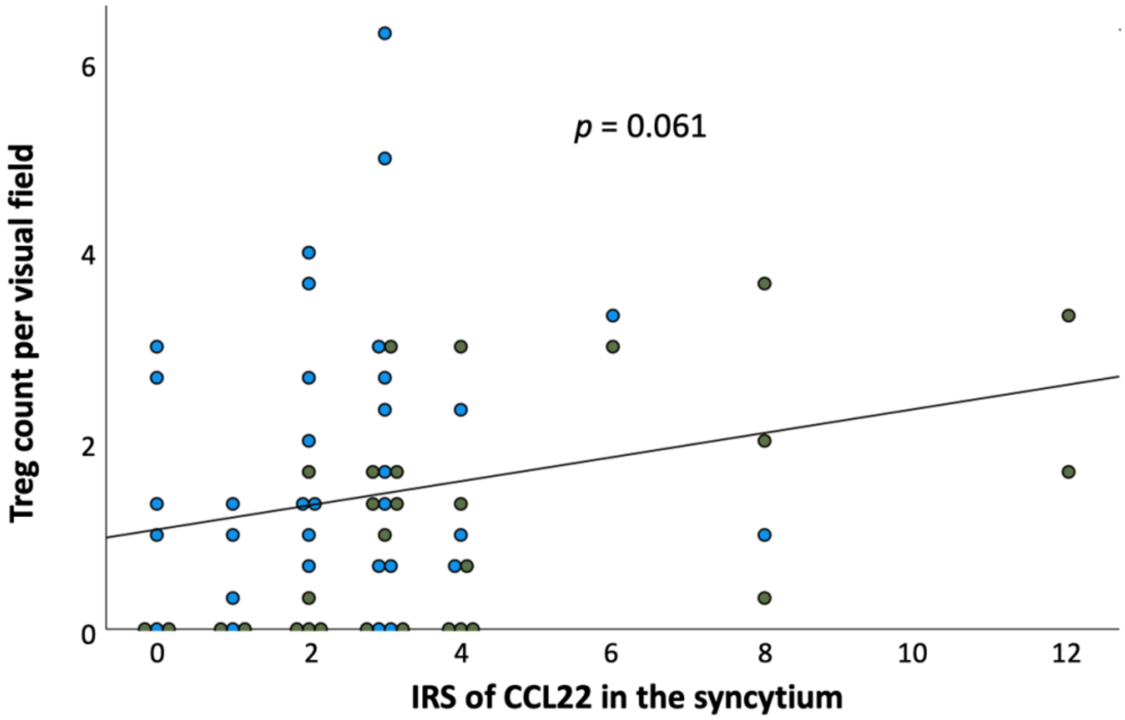

Figure S5. Visualization of the correlation of the expression of CCL22 in the syncytium and the number of Tregs per visual field. Correlation was calculated using the Spearman-Rho-Correlation-Test (r = 0.252, p = 0.044). The regression line refers to the total collective. The 34 control placentas are shown in blue, the 32 PE placentas in green.

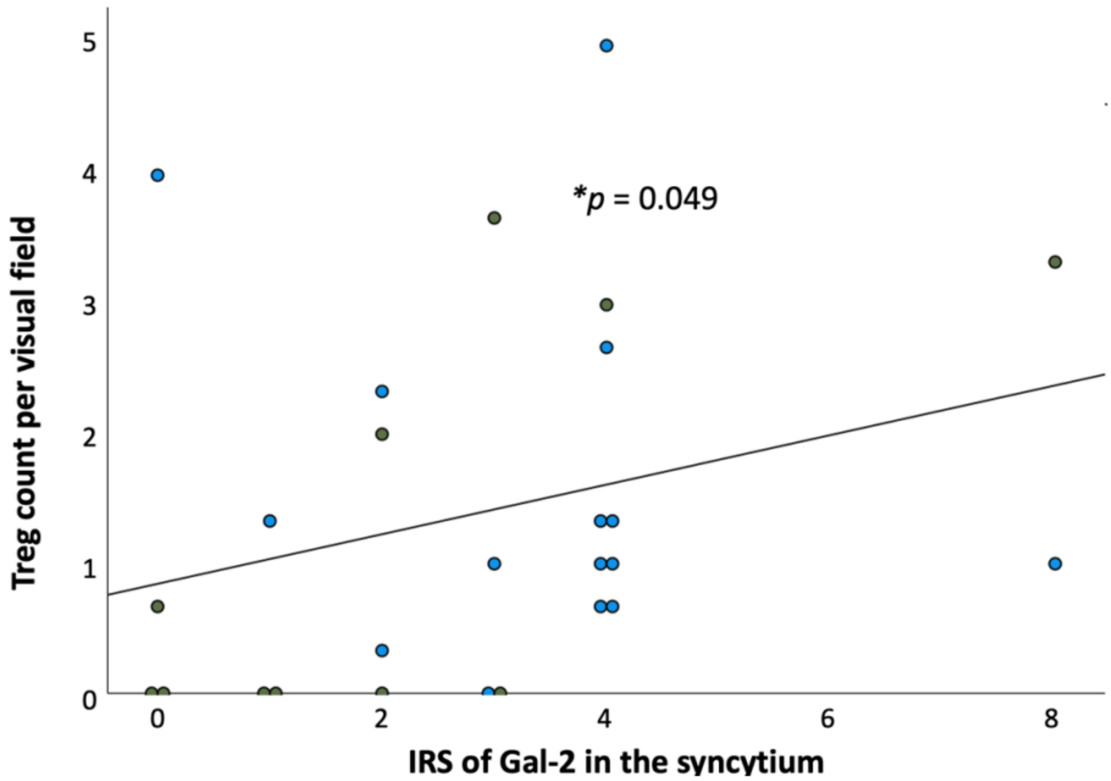

Figure S6. Visualization of the correlation of the expression of Gal-2 in the syncytium and the number of Tregs per visual field. Correlation was calculated using the Spearman-Rho-Correlation-Test ($r = 0.399$, $p = 0.035$). The regression line refers to the total collective. The 15 control placentas are shown in blue, the 13 PE placentas in green.

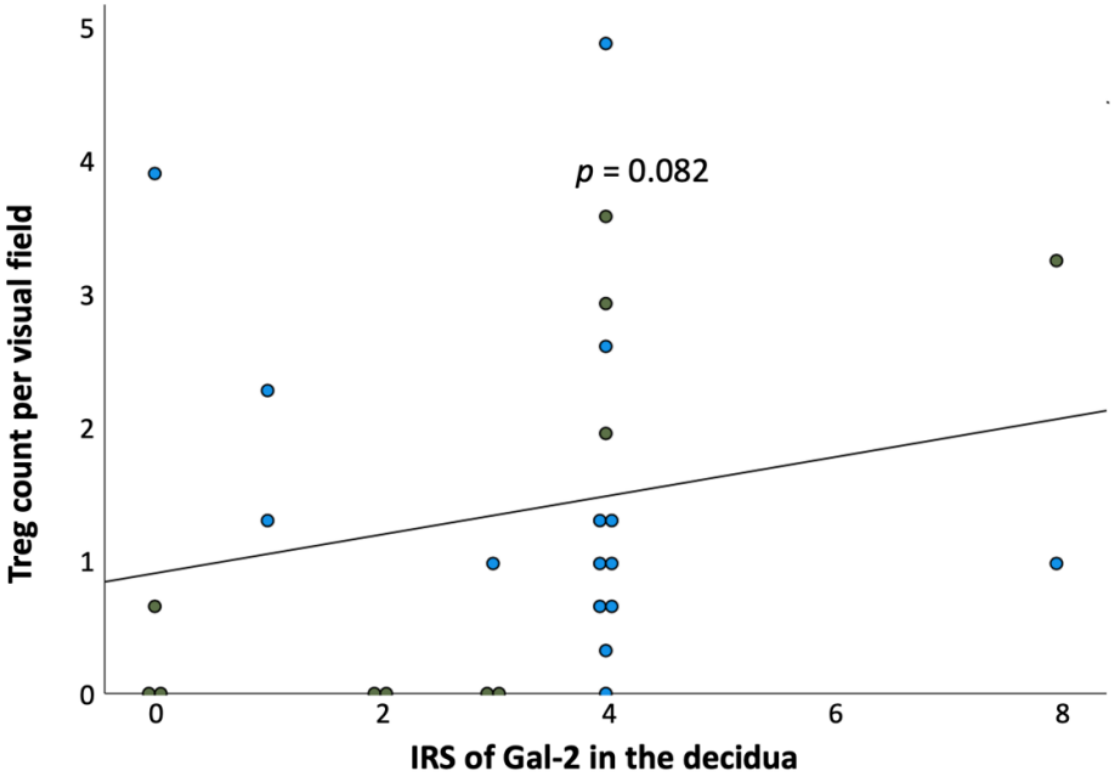

Figure S7. Visualization of the correlation of the expression of Gal-2 in the decidua and the number of Tregs per visual field. Correlation was calculated using the Spearman-Rho-Correlation-Test, the correlation showed to be not significant. The regression line refers to the total collective. The 34 control placentas are shown in blue, the 32 PE placentas in green.

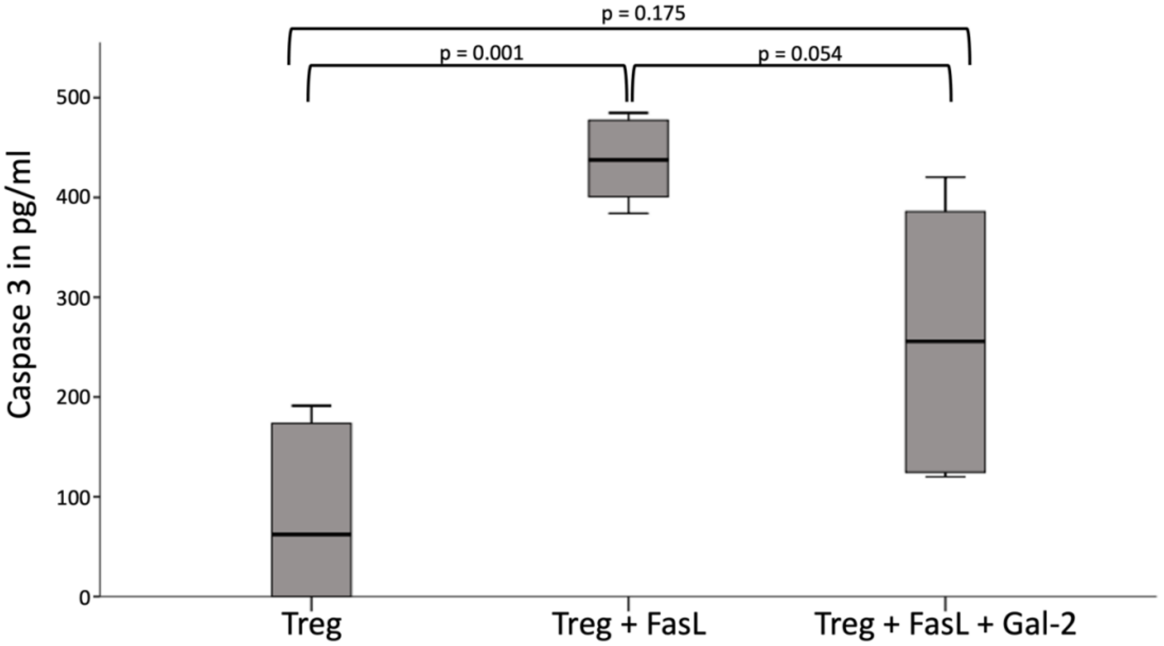

Figure S8. Level of active Caspase 3 after exclusion of the second donor in the groups untreated Treg (Treg, 81.56 ± 91.979), Treg with induction of apoptosis through FasL (Treg + FasL, 437.02 ± 43.915) and the group with the Gal-2 treatment (Treg + FasL + Gal-2, 260.39 ± 147.971) The one-way-ANOVA-Kruskal-Wallis-Test showed significant differences through the three groups ($p = 0.004$). Further analysis revealed this significant difference between the group Treg and the group Treg + FasL ($p = 0.001$).

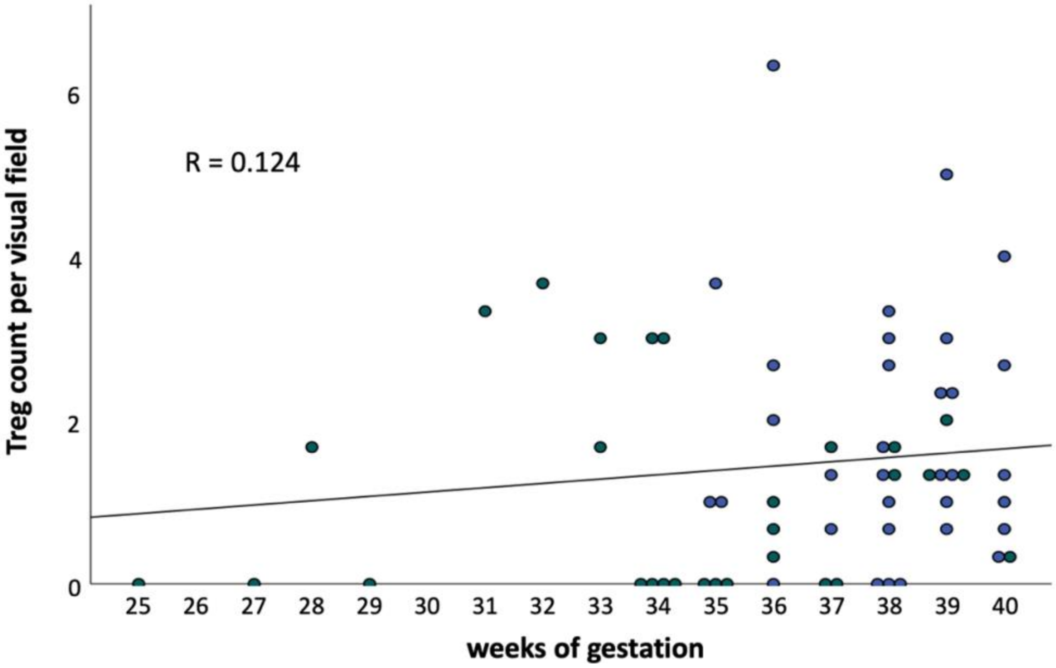

Figure S9. Visualization of Treg count per visual field in relation to the weeks of gestation. The regression line represents no significant correlation ($p = 0.339$). Linear regression was used to analyze the effect of the week of gestation on the Treg count per visual field.

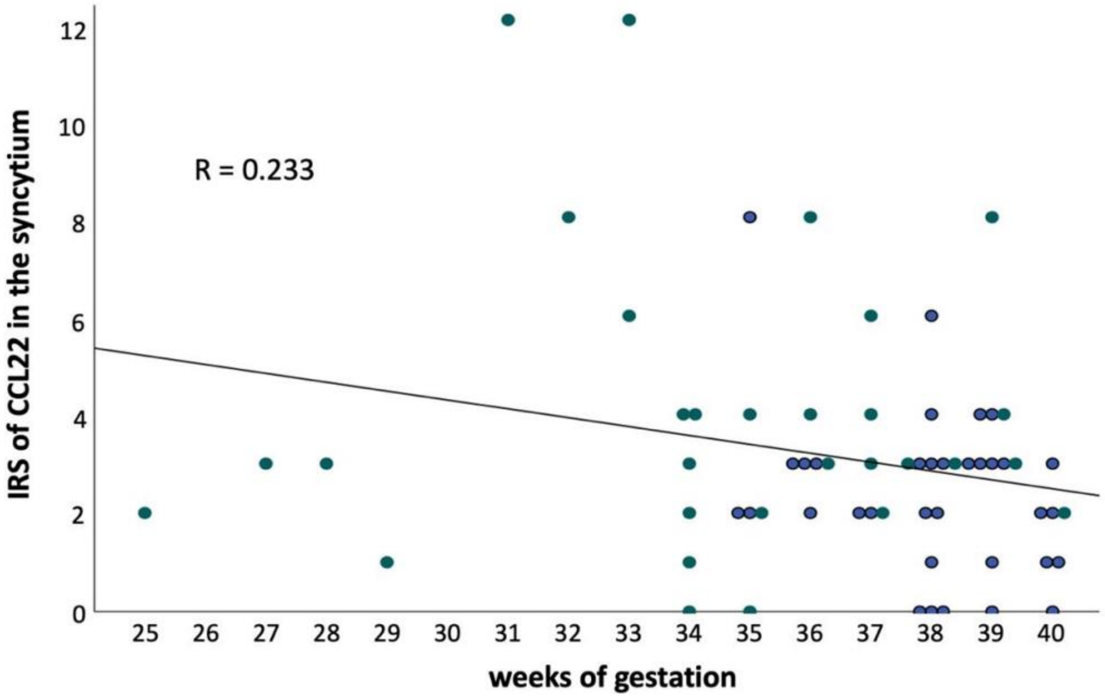

Figure S10. Visualization of CCL22 expression in relation to the weeks of gestation. The regression line represents no significant correlation ($p = 0.064$). Linear regression was used to analyze the effect of the week of gestation on the IRS of CCL22 in the syncytium.

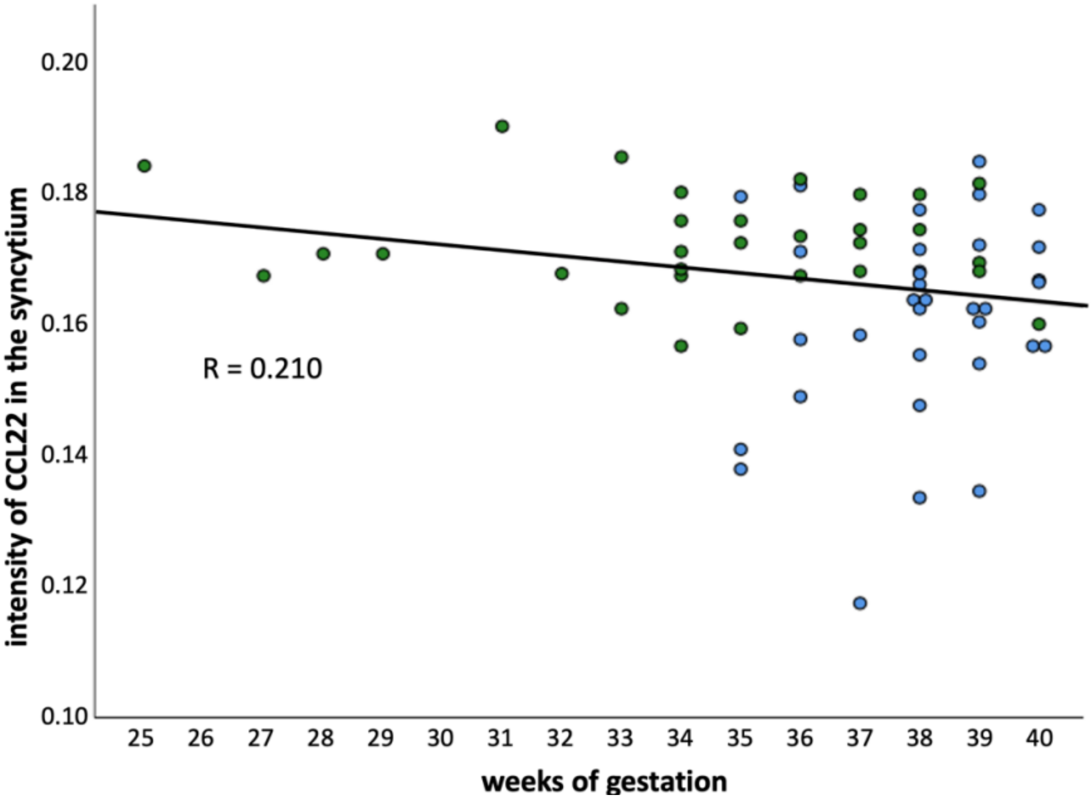

Figure S11. Visualization of the intensity of CCL22 in relation to the weeks of gestation. The regression line represents no significant correlation ($p = 0.096$). Linear regression was used to analyze the effect of the week of gestation on the intensity of CCL22 in the syncytium.

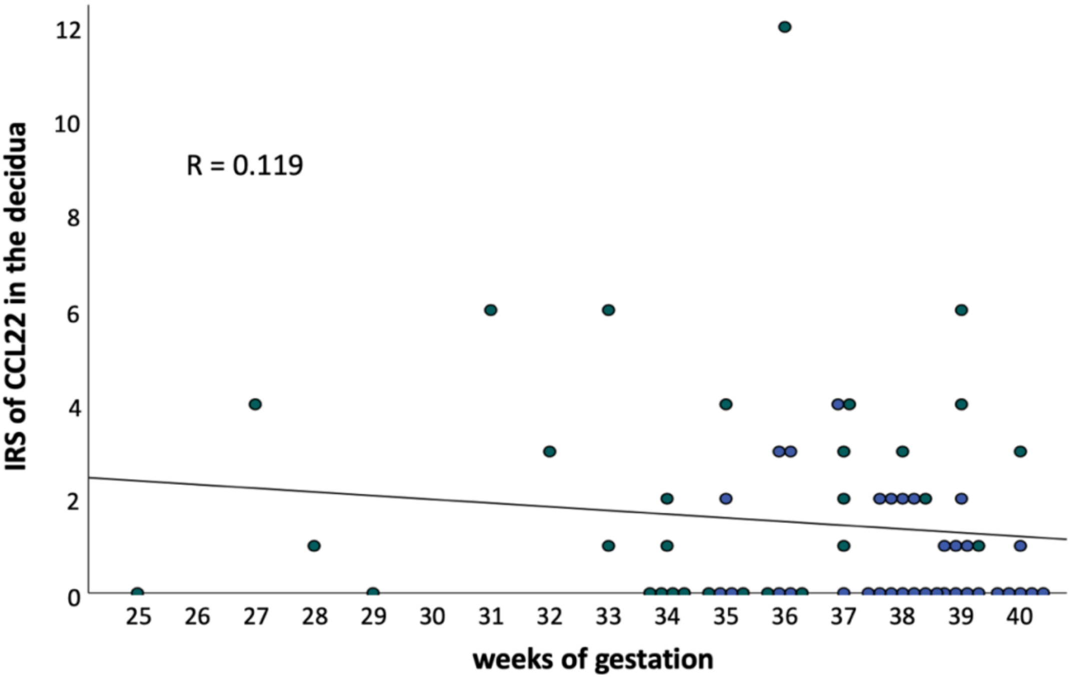

Figure S12. Visualization of CCL22 expression in relation to the weeks of gestation. The regression line represents no significant correlation ($p = 0.350$). Linear regression was used to analyze the effect of the week of gestation on the IRS of CCL22 in the decidua.

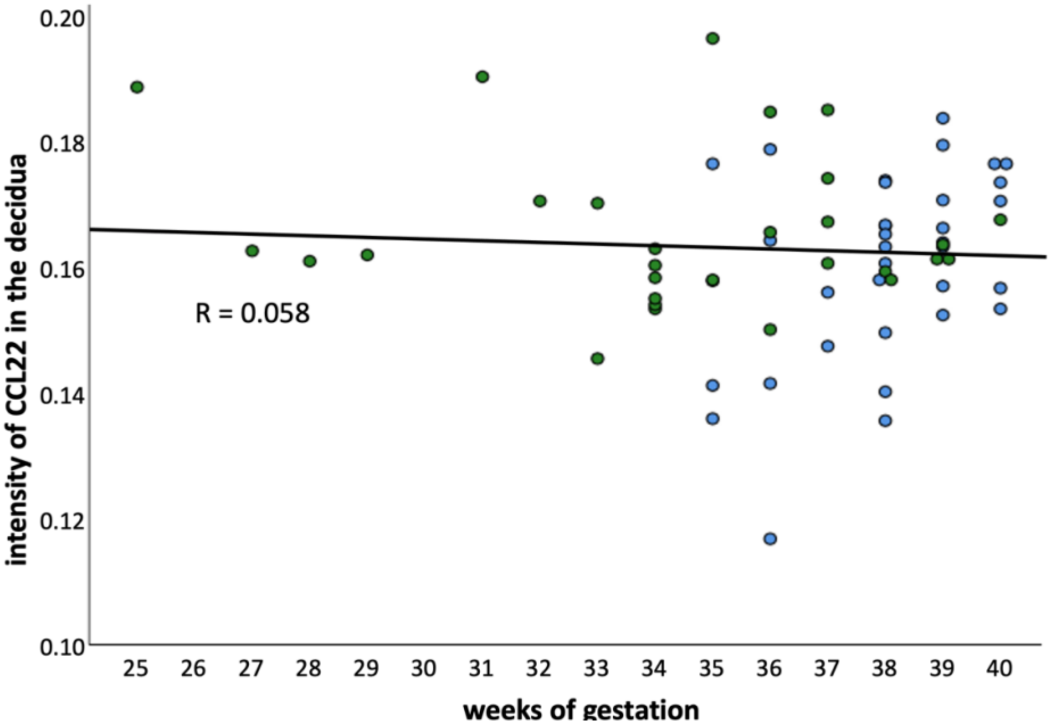

Figure S13. Visualization of the intensity of CCL22 in relation to the weeks of gestation. The regression line represents no significant correlation ($p = 0.648$). Linear regression was used to analyze the effect of the week of gestation on the intensity of CCL22 in the decidua.

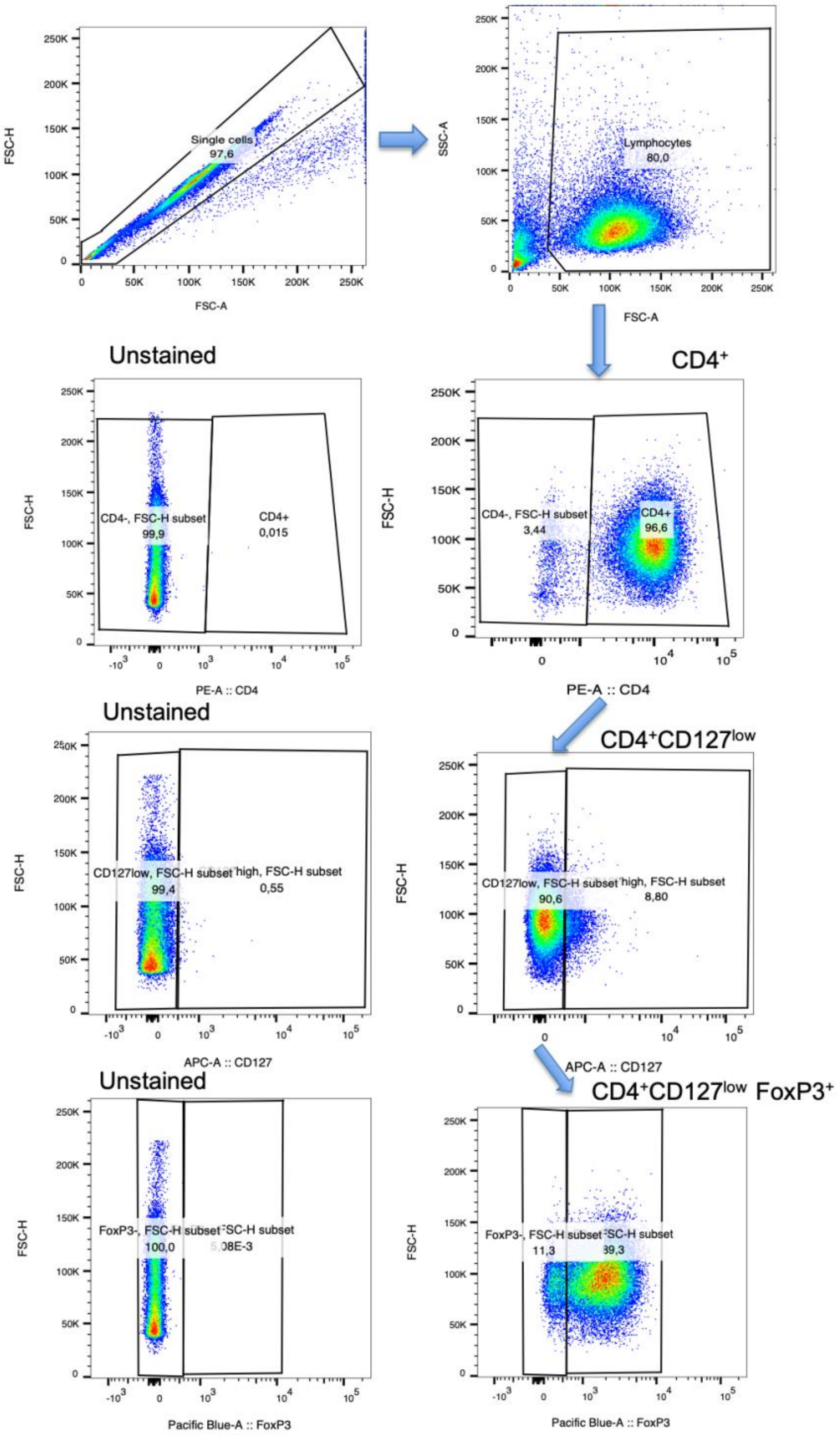

Figure S14. Purity of isolated regulatory T-cells (C4+CD25+CD127low cells). Gating strategy was devel- oped by analyzing unstained PBMCs to set negative gates and apply on the Treg-sample which was obtained after MACS Isolation. Purity of isolated Tregs was confirmed with FACS-staining of corresponding antibodies (Supp. Tab. 2) and amounted to 76% of gated lymphocytes.

Table S1. Gestational age and sex of the babies of healthy and PE affected pregnancies.

Controls					PE						
Week of Gestation	Sex of the Baby	Weight of the Baby (g)	APGAR	Maternal Age	Week of Gestation	Sex of the Baby	Weight of the Baby (g)	APGAR	Maternal Age	Proteinuria (mg/dL)	Blood Pressure (mmHg)
40 + 3	male	-	-	17	40 + 0	male	3110	10/10/10	38	33.7	190/111
40 + 3	male	-	-	25	39 + 2	male	3750	4/7/9	30	82	143/88
40 + 2	female	-	-	39	39 + 2	male	3560	9/10/10	26	300	156/93
40 + 2	female	-	-	31	39 + 0	male	3260	10/10/10	-	300	170/100
40 + 1	female	-	-	33	38 + 0	male	3030	10/10/10	30	-	-
40 + 0	female	3260	9/10/10	35	38 + 0	female	2955	9/10/10	29	300	160/113
39 + 6	male	-	-	30	37 + 6	male	2530	8/10/10	40	-	157/84
39 + 6	female	-	-	23	37 + 5	female	2830	8/10/10	35	72	165/95
39 + 2	male	4000	9/10/10	35	37 + 3	male	2685	8/10/10	38	33.8	155/115
39 + 1	male	3675	9/10/10	39	37 + 2	female	2750	9/10/10	29	-	-
39 + 1	male	-	-	35	36 + 4	male	3250	8/10/10	31	100	-
39 + 1	male	-	-	32	36 + 1	female	2650	3/9/10	31	587	152/77
39 + 0	male	-	-	32	36 + 1	female	2220	5/8/10	31	587	152/78
39 + 0	female	-	-	32	35 + 3	female	2500	9/10/10	39	191	166/88
38 + 5	female	3950	9/10/10	25	35 + 0	female	2330	8/10/10	36	221	170/83
38 + 4	male	2790	9/10/10	41	35 + 0	female	1555	8/10/10	-	-	180/100
38 + 4	female	-	-	37	34 + 6	-	-	-	-	-	-
38 + 4	male	2650	7/8/9	33	34 + 3	male	2100	9/10/10	38	51,4	176/100
38 + 3	female	2875	9/10/10	26	34 + 3	male	1980	10/10/10	38	51,4	176/100
38 + 3	female	-	-	35	34 + 1	male	1704	9/10/10	-	-	-
38 + 3	female	3760	8/8/10	31	34 + 1	female	1735	8/10/10	31	417	180/120
38 + 2	female	3675	9/10/10	39	34 +1	male	2000	7/9/10	-	300	190/100
38 + 1	female	3310	9/10/10	37	33 + 5	female	1770	7/9/9	39	35.2	149/101
38 + 1	male	3330	10/10/10	27	33 + 5	male	1990	4/8/9	27	-	-
38 + 0	male	-	-	22	32 + 5	-	-	-	29	-	-
37 + 0	female	2580	9/10/10	22	31 + 1	Female	1370	7/9/9	40	156	195/95
37 + 0	male	2430	9/10/10	22	29 + 5	-	-	-	-	-	-
36 + 5	male	3130	8/9/10	44	28 + 6	female	930	8/9/10	28	140	155/97
36 + 2	male	3375	7/9/10	34	27 + 1	male	680	8/8/8	29	39.8	170/110
36 + 0	female	2550	9/10/10	31	25 + 6	male	570	4/7/9	33	-	-
36 + 0	female	2260	9/10/10	38	-	-	-	-	-	-	-
35 + 6	female	2640	9/10/10	34	-	-	-	-	-	-	-
35 + 4	male	-	-	34							
35 + 1	female	-	-	38							

Table S2. Antibodies used for the FACS staining.

antibody	species isotype	company	dilution
**APC-CD127**	antihuman	Biolegends 351315	1:100
**APC-CD3**	antihuman	Biolegends 300311	1:167
**FITC-CD25**	antihuman	Biolegends 356105	1:100
**Pac.Blue-CD19**	antihuman	Biolegends 302223	1:167
**Pac.Blue-FoxP3**	antihuman	Biolegends 320215	1:5
**PE-CD4**	antihuman	Biolegends 317409	1:167

Supplementary statistical evaluation of the overall collective

Since the gestational weeks of two PE placentas were missing, we excluded them from the analysis, but decided to present the results of the overall analysis as well, if there is any interest. Since some data related to analyses regarding the weeks of gestation or only the control group do not differ, we report these results only in the main manuscript.

The number of FoxP3-positive cells was significantly reduced (p = 0.036) in PE placentas (1.04 ± 1.180) compared to control placentas (1.80 ± 1.497). - The staining result of CCL22 in the syncytium showed a significantly increased CCL22 cytoplasmatic expression as well as an increased intensity in PE placentas (IRS = 3.81 ± 2.999; intensity = 0.171 ± 0.0082) compared to the control placentas (IRS = 2.38 ± 1.688, pIRS = 0.035; intensity = 0.161 ± 0.0151; pintensity < 0.001).

Furthermore, the mean IRS of the CCL22 staining in the decidual part of the placenta was significantly higher (p = 0.004) during PE (2.31 ± 2.633) compared to healthy samples (0.76 ± 1.130), while the mean intensity-analysis did not show a significant difference between PE (0.166 ± 0.0154) and the control group (0.161 ± 0.0123).

Moreover, the expression of CCL22 in the syncytiotrophoblast and in the decidua correlated significantly positive (r = 0.360, p = 0.003), indicating an increased expression in the entire placenta in the case of PE.

The expression of CCL22 and the number of placental Tregs correlated significantly positive in the syncytiotrophoblast (r = 0.252, p = 0.044) and the EVT (r = 0.256, p = 0.042). Furthermore, individual examination of PE and controls revealed a significantly positive correlation between the number of Tregs and the expression of CCL22 in the syncytium in PE placentas (r = 0.559, p = 0.001).

A significant positive correlation between Gal-2 in the syncytiotrophoblast and the number of decidual Tregs was detected (r = 0.399, p = 0.035). Considering control and PE placentas individually, a significantly positive correlation was shown between the number of Tregs and the expression of Gal-2 in PE placentas (syncytium: r = 0.614, p = 0.026; decidua: r = 0.628, p = 0.022).

Supplementary statistical evaluation of matched data regarding gestational week

The data was analyzed using the non-parametric Wilcoxon-Rank-Test since a normal distribution cannot be assumed. While there was no significant difference regarding the expression of CCL22 in the syncytium, despite descriptive differences which can be seen in the table above, significant differences were found for the expression of CCL22 in the

EVT (p = 0.028) as well as for the average number of Treg (p = 0.046). Furthermore, considering the table presented above, it can be seen that the matched data show the same trend as the overall collective, meaning a lower expression of CCL22 but more Treg in the controls than the preeclampsia placenta. Moreover, not only the IRS but also the quantitative measurement of CCL22 follows the trend shown by the whole collective. While no significant difference between the intensity of CCL22 in the decidua could be detected, the significant difference of the intensity of CCL22 appears also when only the matched data is analyzed (p = .046).

Table S3. Mean-value ± SD of the IRS of matched data used for the Wilcoxon-Rank-Test.

Week of gestation		IRS of CCL22 in the syncytium	IRS of CCL22 in EVT	Count of Treg
40	Control	1.50 ± 1.049	0.17 ± 0.408	1.67 ± 1.399
	PE	2.00	3.00	0.33
39	Control	2.63 ± 1.408	0.63 ± 0.744	2.12 ± 1.402
	PE	5.00 ± 2.646	3.67 ± 2.517	1.55 ± 0.387
38	Control	2.18 ± 1.888	0.73 ± 1.009	1.37 ± 1.271
	PE	3.00 ± 0	2.50 ± 0.707	1.50 ± 0.240
37	Control	2.00 ± 0	2.00 ± 2.828	1.00 ± 0.467
	PE	3.75 ± 1.708	2.50 ± 1.291	0.56 ± 0.964
36	Control	2.75 ± 0.500	1.50 ± 1.732	2.75 ± 2.642
	PE	5.50 ± 3.536	6.00 ± 8.485	0.67 ± 0.474
35	Control	4.00 ± 3.464	0.67 ± 1.155	1.89 ± 1.542
	PE	2.00 ± 2.00	1.33 ± 2.309	0.00 ± 0.000
